# National and transnational drug shortages: a quantitative descriptive study of public registers in Europe and the USA

**DOI:** 10.1186/s12913-022-08309-3

**Published:** 2022-07-22

**Authors:** Reko Ravela, Alan Lyles, Marja Airaksinen

**Affiliations:** 1 Clinical Pharmacy Group, Division of Pharmacology and Pharmacotherapy, Faculty of Pharmacy, University of Helsinki, Helsinki, Finland; 2 School of Health and Human Services, College of Public Affairs, University of Baltimore, Baltimore, USA

**Keywords:** Drug shortage, Medicine shortage, National drug shortage registers, Public registers, Open data

## Abstract

**Background:**

Drug shortages are a growing global problem, posing clinical and economic challenges. To understand them better, we conducted an inventory of national public drug shortage registers and their comparability in Europe and the USA.

**Methods:**

The study was based on openly accessible drug shortage notifications published by national drug authorities. These data were obtained from all national data sources mentioned on the European Medicines Agency’s (EMA’s) web page and FDA in the USA. After selection of the countries with comparable data, descriptive statistics were used to present characteristics of the shortages both across countries and within countries for 9 months (January–September) in 2020. We studied whether the shortages that occurred in these countries were the same, and how shortages were distributed by therapeutic uses and formulations. We also investigated price variation between the United States and Finland among drugs in shortage in one formulation category (creams and gels).

**Results:**

Finland, Sweden, Norway, Spain, and the United States had suitable registers and were included. Altogether 5132 shortage reports from Finland (*n* = 1522), Sweden (*n* = 890), Norway (*n* = 800), Spain (*n* = 814), and the United States (*n* = 1106) were published during the study period. Of active ingredient level shortages 54% occurred in only one country, and 1% occurred in all five. However, at the country level, where there was one or more shortage notifications in an ATC active ingredient category, 19–41% were in a single country.

The distributions by ATC therapeutic class and drug formulation differed substantially between countries, particularly between the USA and European countries. Injectables had a high shortage risk in the USA (57% of all shortages versus 17–31% of all shortages in the European countries). By contrast, shortages in gels and creams occurred only in European data (4–6% of all shortages). In the price comparison, creams and gels in shortage in Finland were 160% more expensive in the USA where these shortages were not detected.

**Conclusions:**

Public drug shortage registers are vital data sources for proactively maintaining and managing a reliable drug supply. However, our study demonstrates that much work remains to standardize the contents and quality of public register data.

Shortages may not be solely a consequence of manufacturing disruptions but may reflect other contributing factors in the international drug distribution and supply mechanisms, including price differences and profit margins between national pharmaceutical markets. Data to perform practical and useful international comparisons to understand these shortages are required.

**Supplementary Information:**

The online version contains supplementary material available at 10.1186/s12913-022-08309-3.

## Background

Drug shortages pose clinical and economic challenges for health care systems globally [1–3]. Shortages may delay or cancel treatments [4–6], increase medication errors [7], and cause added costs for healthcare systems and societies [1, 8, 9]. From a patient perspective, they mean extra cost and hurdles to get needed medications [10, 11], increased insecurity, and decreased quality of life [12]. In the worst cases, drug shortages can prevent effective care, increase hospitalisations, and even increase mortality [5, 6, 9, 12].

Published statistics and surveys show that drug shortages have increased in many, if not most European countries since 2010 [1, 13–19]. In a 2019 survey of European hospital pharmacists [18], 95% of respondents considered drug shortages a current problem in delivering the best care to patients and 63% reported that drug shortages had an impact on patient care in their hospital.

However, shortage registers have mainly been used in national level research and thus far cross-country research of register data has been limited. Such contrasts require harmonized definitions, and only recently, there is an emerging consensus among EU countries, on what constitutes a drug shortage and how they should be communicated [20–22].

In 2019 the European Medicines Agency (EMA) published its guidance on a common definition for what constitutes a drug shortage and provided guidelines for communicating these shortages to the wider public [23]. As supply problems have increased, many national authorities have increased their emphasis on registration and publication of shortages. The present study is the first, to our knowledge, (i) to combine and compare register data under the new EU guidance, (ii) to compare European data to shortage notifications published by the United States Food and Drug Administration (FDA), and (iii) to present shortage data spanning the start of the coronavirus pandemic.

## Methods

In most European countries and in the United States, market authorisation holders are obligated to notify national authorities of impending drug shortages [20, 24]. Many countries also publish these notifications, even if they only contain partial information. Our study is based on this kind of public data, i.e., openly accessible drug shortage notifications published by national drug authorities. These data were obtained from all of the national data sources mentioned on the European Medicines Agency’s (EMA’s) web page [25]. Extraction of data was done by one of researchers (RR) under supervision of another (MA).

This study charted available data from national public shortage registers individually and in the aggregate. The consolidated data gave quantitative insights on drug shortages and hints on their underlying causes in the selected European countries and the USA. Aspects of special interest included the number of shortages documented in the register within the study period, specific drugs in shortage, their therapeutic category as defined by WHO ATC coding/classification [26] and their formulation. To explore the possible contribution of product prices to shortage differences between countries, we compared retail drug prices for creams and gels between the USA and Finland.

The resulting research database included drug shortage notifications made during the first 9 months of 2020 (January 1–September 30, 2020). In cases where both the notification date and starting date of the shortage were found, cases were included and excluded based on the notification date. If only the starting date or notification date were mentioned, the date available was used as the inclusion criteria.

Notifications that announced a product discontinuation from the national market were excluded. Cases where “market considerations” was given as the reason for the drug shortage were considered an announcement of a planned discontinuation by the drug company and were, therefore, excluded. Notifications concerning veterinary drugs were also excluded.

In addition to national shortage registers, Finland’s national drug register maintained by the Finnish Medicines Agency (Fimea) [27] was used to perform a more in depth, intra-country analysis for risk(s) of drug shortage(s) by ATC main class and drug formulation.

The Anatomic-Therapeutic Chemical (ATC) classification system maintained by WHO is based on a five-level hierarchy (Table 1). In our analysis we used the highest level, which divides medications into the main classes and the lowest level, which specifies active ingredient(s). For clarity, we refer here to the highest level as the ATC main class and the lowest level as the ATC ingredient category.

**Table 1:** Example of WHO’s Anatomic-Therapeutic Chemical (ATC) classification with nervous system medication [26]

ATC Category	Label	Description
N	Nervous system	Main class level
N05	Psycholeptics	
N05C	Hypnotics and sedatives	
N05CD	Benzodiazepine derivatives	
N05CD08	Midazolam	Ingredient category level

### Statistics and research ethics

Descriptive statistics were used to present the distribution of shortages by ATC main class and drug formulation both across countries and within countries. In the analysis of Finnish shortages, differences in distribution of shortages vs. all marketed drugs were evaluated using the z-test. Analyses were performed using IBM SPSS Statistics for Windows version 26.

The registers did not include any patient data. As material in this study consisted of openly accessible retrospective register data, consequently, research permits, or ethics committee evaluation were not needed. Appropriate standards of scientific practice and research ethics were followed throughout the study [28].

## Results

### Selection of countries

**Fig. 1:**
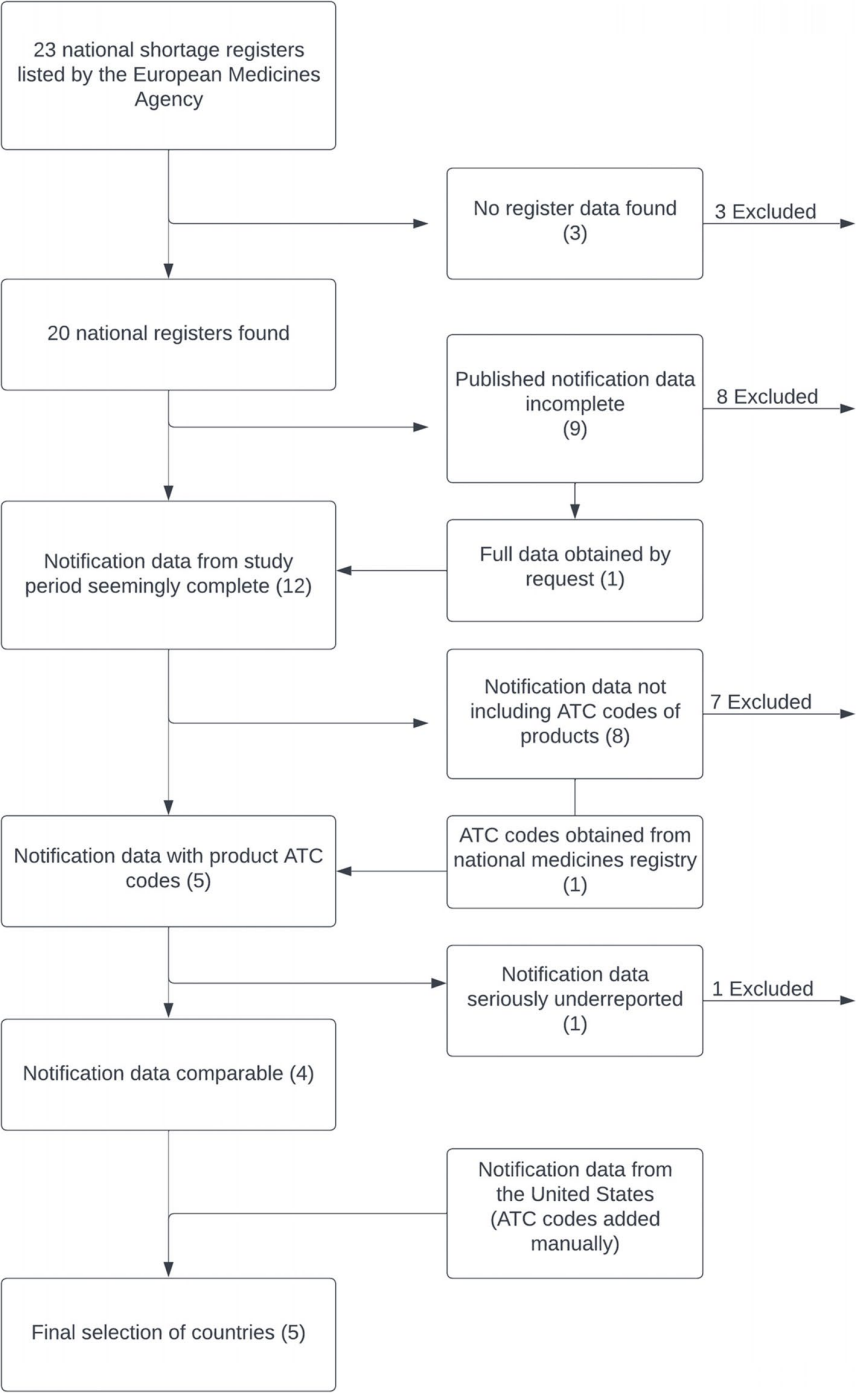
Selection of countries in the study

Four European Economic Area (EEA) countries (Finland, Sweden, Norway, Spain) and the USA had suitable registers and were selected from an inventory of publicly available shortage notification data (Fig. 1).

The EMA listing [25] identified 23 national drug authorities in October 2020 (Appendix 1). All of these public notification shortage registers were reviewed between the 1st and 16th of October 2020. In three of these, shortage data could not be found or accessed at all. Eight of the remaining 20 countries were then excluded because their published data were incomplete – covering only part of all notified shortages during the study period. Finnish shortage register data found on the web also only included current shortages, not shortages from earlier time periods, though the full data from the research period was received by request from Finnish Medicines Agency. Next, seven countries were excluded because there were no ATC codes signifying active ingredient(s) and use. In one case (Spain) this issue was solved by combining ATC codes to data by using the national drug registry available at Spain’s same web page.

One country (Hungary) was excluded because number of notifications in the register was dramatically smaller than in other registers which were included, and so may have distorted the findings. Since other sources [17, 18] did not provide evidence that actual shortages were more rare in Hungary, we determined that the data was not considered reliable enough to be representative of current realities.

Decisions to include Finland, Spain and the United States were made on reasoning that a strictly systematic approach would have limited the study to only two countries with comparable registers (Sweden and Norway).

Finnish data was specifically requested from Finnish Medicines Agency based on our purpose of comparing the situation in Finland with other countries. The data obtained are included in the present study.

Data from Spain was included to provide a contrast with a larger, non-Nordic drug market within the EU/EEA. Spain’s shortage register database was included as it represented the most comprehensive among the top 5 European markets (France, Germany, Italy, Spain, and the United Kingdom) and issues with the lack of ATC codes could be solved by using Spain’s own national medicines registry [29], which was available.

Shortage data from the USA published by the US Food and Drug Administration (FDA) [30] was also included because it is an important reference point, despite differences in the comprehensiveness of its shortage register (Table 2). USA data, which included active ingredients, but not ATC classification, was complemented with the help of an ATC code search engine on a WHO web page [26].

**Table 2:** A comparison of USA and Finnish drug shortage registers

	Point of notification	Shortages created by logistics or demand	Drugs included	Published registry
Food and Drug Administration FDA, USA	Manufacturing disruption	Voluntary notification	Prescription drugs	Publication by national authority based on significance for supply
Finnish Medicines Agency Fimea, Finland	Supply disruption	Obligatory notification	All registered drugs	Direct publication of notifications

Source(s): Finnish Medicines Agency Fimea [31, 32], US Food and Drug Administration FDA [24, 30]

Register data structures differed substantially between countries. Therefore, we compared reporting differences between the USA and Finland as an example (Table 2) using guidance papers published by their national authorities [24, 31]. Although national guidances in EU countries may vary, they should be all based on EU guidelines [23], as is the case with Finland.

USA data did not have a date of notification, and the starting date was found in only a small subset of cases. However, in all cases the date of the last update was available. Both starting date and date of the last update were used as exclusion criteria if they were outside the study’s time period.

Over the counter (OTC) drugs were included in data from all of the European countries included in the study but was not available in the USA data (Table 2). In intra-country analysis, 11.4% (*n* = 174) of Finnish cases of shortages (*n* = 1522) were OTC drugs, while 9.8% of all marketed drugs in the national registry were OTC products (Table 5). Shortage registers as such did not include data about the prescription status of products. Complete exclusion of OTC drugs from the study would have required searching for this information for all products, which was considered logistically infeasible at this point.

In the end, notifications, referred to here as cases, from four European countries (Finland, Norway, Sweden, Spain) and the USA were included in the study. There were altogether 5132 shortage notifications: 1522 from Finland, 890 from Sweden, 800 from Norway, 814 from Spain and 1106 from the USA.

### National or transnational shortage?

Using product ATC classifications [26], we found shortages in a total of 893 ATC ingredient level categories (Table 3). These ATC categories were then analysed to determine whether shortages in the same ingredient category occurred in only one country (that would be a ‘unique shortage’), more than one country, or in all countries in the study.

**Table 3:** ATC ingredient categories that contained at least one shortage notification, by country during January – September 2020 [30, 32–35]

	Spain	Finland	Norway	Sweden	USA	All Countries
**Number of ATC ingredient categories with at least one shortage**	335	485	297	368	99	893
**Cases, n**	814	1522	800	890	1106	5132
**Cases/category**	2.4	3.1	2.7	2.4	11.2	5.7
**Unique shortages, n**	123	180	56	78	41	478
**Unique %**	36.7%	37.1%	18.9%	21.2%	40.6%	53.5%

There were sizable differences in the unique shortages, meaning shortages in the ingredient category that occurred in only one of the countries being studied (Table 3). The USA had the highest proportion of unique shortages (40.6%), and Norway had the lowest (18.9%).

While the USA had a similar level of supply disruptions in drug products, they were concentrated in fewer ATC ingredient categories than were those in Europe (Table 3).

There was a shortage recorded in all five countries for just nine ATC ingredient categories, while 478 shortages, 53.5% of the total (*n* = 893), occurred in only one of the countries (Tables 3 and 4). The nine drugs in shortage in all five countries during the study period were (in order of ATC code): sulfasalazine (A07EC01); adrenaline (C01CA24); furosemide (C03CA01); diltiazem (C08DB01); erythromycin (J01FA01); propofol (N01AX10); morphine (N02AA01); midazolam (N05CD08); and sertraline (N06AB06).

**Table 4:** Incidence of drug shortages in the study countries by ATC ingredient categories

Number of countries with at least one shortage notification of the same ingredient category drugs						
	5 countries	4 countries	3 countries	2 countries	1 country	Total
**Number & % of ingredient categories**	9 1.0%	61 6.8%	127 14.2%	218 24.4%	478 53.5%	893 100.0%

### Distribution of shortages by ATC main class and drug formulation

**Fig. 2:**
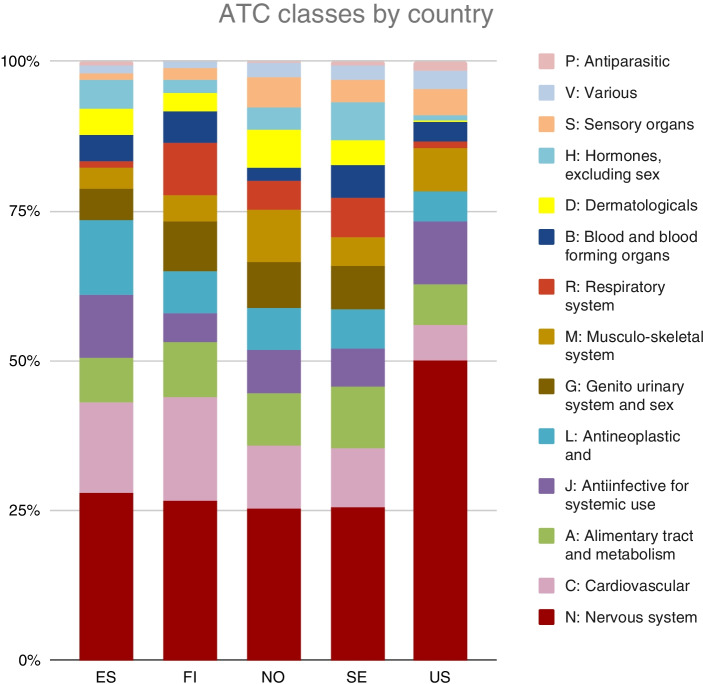
Distribution of shortage notifications (*n* = 5132) according to ATC class and country during January–September 2020

As the cases were sorted by ATC main classes, the highest number of shortages were found in drugs affecting the nervous system (ATC code N): 31.5% (*n* = 1616) of all shortages (*n* = 5132) (Fig. 2). The shortages under this category included anaesthetics, analgesics, sedatives, and psychotropic drugs.

According to ATC main classes, some therapeutic classes had meaningful differences in shortages between study countries (Fig. 2). The USA had a much higher proportion of nervous system drug shortages (ATC code N, 50.0% of all shortages, 553 cases) and practically no shortages of dermatologicals (ATC code D) or sex hormones (ATC code G). Spain, by contrast, had a higher proportion of shortages in antineoplastic drugs (ATC code L, 12.5%, 102 cases), while Finland had an even higher absolute number of shortages of antineoplastic drugs (108 cases).

**Fig. 3:**
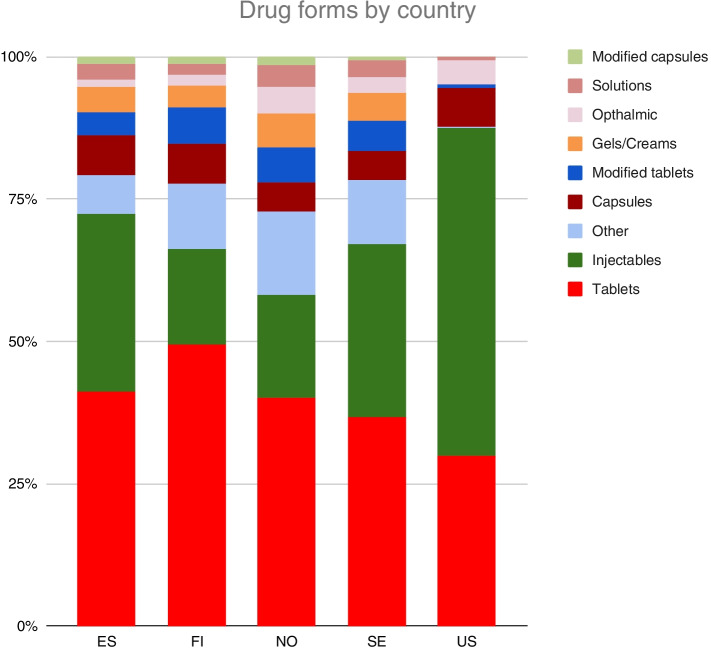
Distribution of shortage notifications (*n* = 5132) by drug formulation and country during January–September 2020

Analysis of distribution by drug formulation showed some clear differences between shortages in the USA versus Europe (Fig. 3). In the USA, shortages were heavily concentrated in injectable products (57.4% of USA shortages) with few cases of oral modified-release products or creams and gels. The category “Other”, which included products such as inhalers, suppositories, and transdermal patches, was also almost non-existent in USA shortage data compared to that in European countries.

In European countries, there were between 37 to 60 shortage cases per country for creams and gels; in the USA, there was none (Fig. 3). A brief examination of price differences between the USA and Finland showed an average 160% higher price for these products in the USA (Appendix 2).

### Comparison of the Finnish national drug shortage registry to active marketing authorisations

To analyse whether certain types of drugs have a supply problem, the Finnish drug shortage register was compared to the national drug register of active marketing authorisations in Finland (Table 5).

**Table 5:** Comparison of Finnish drug shortage notifications (*n* = 1522) and active products in the national drug register (*n* = 8728) by ATC main class, drug formulation, and prescription status during January–September 2020

ATC class	(A) Shortage notifications		(B) Products in drug register		
	n	N %	n	N %	*p*-value^*^
N: Nervous system	406	26.7%	2073	23.8%	0.014
C: Cardiovascular	264	17.3%	1190	13.6%	< 0.001
A: Alimentary tract and metabolism	140	9.2%	785	9.0%	NS
R: Respiratory system	133	8.7%	483	5.5%	< 0.001
G: Genito urinary system and sex hormones	124	8.1%	571	6.5%	0.022
L: Antineoplastic and immunomodulating	108	7.1%	905	10.4%	< 0.001
B: Blood and blood forming organs	79	5.2%	638	7.3%	0.003
J: Anti-infective for systemic use	72	4.7%	667	7.6%	< 0.001
M: Musculo-skeletal system	69	4.5%	407	4.7%	NS
D: Dermatologicals	46	3.0%	262	3.0%	NS
H: Hormones, excluding sex hormones	33	2.2%	172	2.0%	NS
S: Sensory organs	30	2.0%	192	2.2%	NS
V: Various	16	1.1%	363	4.2%	< 0.001
P: Antiparasitic	2	0.1%	20	0.2%	NA
**Drug formulations**					
Tablets	752	49.4%	3755	43.0%	< 0.001
Injectables	258	17.0%	2037	23.3%	< 0.001
Capsules	105	6.9%	739	8.5%	0.040
Modified tablets	97	6.4%	489	5.6%	NS
Gels/Creams	60	3.9%	265	3.0%	NS
Solutions	31	2.0%	259	3.0%	0.043
Ophthalmic	27	1.8%	168	1.9%	NS
Modified capsules	18	1.2%	135	1.5%	NS
Other	174	11.4%	881	10.1%	NS
**Prescription status**					
OTC products	174	11.4%	859	9.8%	NS
Prescription products	1348	88.6%	7869	90.2%	NS
**Total**	**1522**		**8728**		

*NS* Not significant, *p* > 0.05

*NA* Not applicable, sample size too small

^*^comparison of column proportions with z-test

There were several statistically significant differences between shortages compared with all marketed drug products (Table 5). This information, combined with cross-country comparisons, revealed that anti-infectives, hematological and cancer drugs (ATC Classes J and L) were less likely to be in shortage, while cardiovascular drugs, respiratory drugs and sex hormones (ATC Classes R and G) were more at risk of shortage. Nervous system drugs were also found to be slightly overrepresented in shortages even in Finland, although Finland had a lower-than-average proportion of cases for nervous system drugs compared to other countries (Fig. 2). Prescription status did not significantly influence the shortage rate.

According to the drug’s formulation, injectables, capsules and solutions were underrepresented in shortages during the study period, while tablets were overrepresented.

## Discussion

Being the largest cross-country comparison case study of drug shortages so far, these results show that open data provided by public shortage registers can be a valuable source of information to perform research on drug shortages. Although there are issues of data incompatibility and incomplete information in registers, their use was demonstrated to be both feasible and informative.

Improved data gathering by national authorities and other shortage register providers, combined with harmonization of databases and accessibility could enable larger, more accurate and timely analyses. These would, in turn, support timely future policies and managerial decisions. Understandably, many national medicine authorities focus on the mitigation of shortages when they publish data. Not all publish these data. However, to fully understand the dynamics of drug shortages, all shortage notification data should be made publicly available as there appears to be no valid reason for restricting access. Transparency would expedite research and international cooperation to tackle the issue, with decreasing drug shortages improving access for all.

Our findings can be used to support improvement of comparability of data in national registers to better inform public policies for managing shortages. Earlier studies that have tried to characterise drug shortages with register data have largely been conducted on a national level [13, 15, 36–38]. Although this is a valid approach, results in the present study show that caution should be used in generalising these results from one country to situations in other countries.

There are some studies that have previously tried to compare shortage experiences between countries. An earlier study of shortage registers between 2010 and 2013 managed to collect only 671 cases from seven European countries, 66% of them being from a single country, Italy [39]. This amount of data was obviously insufficient and reflected the limited utility of shortage registers at that time.

A recent report published by the European Commission [19] managed to gather 104,507 shortage notifications from 22 European countries between 2007 and 2020 and combine these into a common database. This very broad material is interesting, but uneven, suffering from reporting biases and potentially by biases created by the matching process used. The report’s results, however, are similar to those in our study. Using a different methodology, the European Commission report finds that shortages occur unevenly in different countries and concludes that there are “some fundamental issues that have little to do with sourcing and manufacturing and much more with commercial decisions by suppliers on the one hand and national policies on the other.”

Two previous studies have compared drug shortages in different countries at the hospital level [40, 41]. Interestingly, one of the main results in both has been that while shortages are a common problem in hospitals regardless of the country studied, there is great variation in the specific drugs affected. A 2021 comparison between USA and Australian drug shortages also noted that only 4–7% of drug shortages were the same, despite Australia being heavily dependent on importing drugs from the USA [42].

When taken together with international data, our findings indicate that shortages are more country-specific than transnational. There were significant divergences in both the nature and extent of shortages between the USA and European countries. As a larger number of products in the category having supply problems implies a potentially more serious shortage, concentration of notified product shortages in fewer ATC ingredient categories in the USA might at least partially reflect the higher threshold for publishing shortages there.

Packaging requirements differ across national markets and this might create some of the bottlenecks. International logistical issues might also have some contribution. Neither of these issues should be overlooked, but the main explanations for national differences appear to reside elsewhere.

In the Finnish drug register there were 1397 ATC categories with actively marketed products during the study period. More than 60% of these drugs had at least one product shortage in some of the the five countries investigated. 17,4% of drug products in Finland experienced shortage in the nine -month study period. In this perspective, shortages of drug products are not exceptional, but rather a common phenomenon.

Most of these product shortages are of minor importance, since usually there are generic alternatives available. Especially when there is timely notification of an impending drug shortage, other marketing authorisation holders can increase their production or imports can be increased. Since truly global shortages are rare, this can usually resolve the situation.

According to earlier research and reports [3, 15, 38, 43], shortages are concentrated in low-priced generic products, so for this sector of the drug market, shortages are more normative. Despite low prices, some of these drugs might nonetheless be critical for patient care.

However, unreliability of supply increases the risk of more serious shortage(s), where all alternative products may be out of stock and availability of the drug is completely blocked. Since markets of most individual drugs are oligopolies with only a few providers, the risk of a true shortage is quite real. In Finnish markets there are on average 6,2 products per ATC ingredient category, including different strengths from same supplier. Many generic drugs only have two or three products. The concentration suggests that stock unavailability is a problem waiting to happen under current market conditions.

As seen in the cross-country comparison and in earlier published studies and reports [2, 44–46], the USA shortage problem has been disproportionately concentrated in generic injectables, and in nervous system drugs. There were also clear differences in other therapeutic classes and drug formulation categories - especially between USA and European countries, though also among European countries.

No global pattern of drug shortages was found with the factors investigated, despite the onset of a global COVID-19 pandemic during study period. These observations suggest the influence of differences in national drug market structures on drug shortages that should be studied more in detail in future studies.

In earlier USA studies, injectable products were found to be at especially high risk of shortage [3, 44, 46]. Our findings are in line with these studies. However, this does not seem to be the case in Finland nor in other European countries - injectables form a smaller share of shortages there than in the United States. Neither price nor the generic status of drugs was investigated in our study, since this was not mentioned in any of the registers studied.

Reflecting the pandemic situation during the study period, three (propofol, morphine and midazolam) of the nine drugs which were in shortage were needed by all study countries for intensive care of COVID-19 patients. Most shortages, however, occurred in only one country, underlining the need to see shortages as an outcome of an inequitable and ineffective international distribution of the drugs, rather than solely a manufacturing issue.

There are studies linking drug shortages to economic factors [3, 38, 46]. Differences in shortages found in the present study are plausibly due in part to economic factors. In the USA, 57% of shortages consisted of injectable products, whereas in Finland they were only 17%. A plausible explanation lies in the injectables market structure, including the concentration of manufacturers and low profit margins for specific products.

Injectables are mainly used in hospitals and bought through tender contracts. In Finland, with a population of 5.5 million, there are five hospital purchasing organisations [47]. In the USA, a country of 330 million inhabitants, there exists a handful of large healthcare purchasing organisations. They each represent thousands of hospitals and billions of dollars in purchasing power [48]. Independent verification of prices is impeded as hospital contracts are typically confidential. Since USA purchasing organisations have substantial purchasing power, they can likely push prices of generic injectables to lower levels than those in Finland. However, without sufficient mechanisms to guarantee supply, low prices can increase injectable shortages as international drug companies see the USA market for generic injectables as a low priority.

Similarly, a lack of shortages affecting creams and gels in the USA can be explained by price and market size differences. In this situation, Finland is a low priority market and shortages are seen there, but not in the USA.

These kinds of economic mechanisms seem especially relevant considering most drug shortages are not global but manifest themselves in only one or few countries while other countries are unaffected.

### Study limitations

A broader analysis of price correlation would certainly be an interesting and useful line of inquiry for future research. However, our pilot analysis of prices showed that there are impediments to this type of analysis, as reliable price information is not reliably available and when it is, price can have a different meaning within and across the same market’s segments. This is especially for the hospital medication market, where actual pricing is generally confidential. We would have liked to make this kind of comparison at least for injectables, which also seemed to have very clear differences in shortages between countries, but for the reasons mentioned above, found it beyond our means at the moment.

The material available for our research did not allow us to distinguish between shortages for brand versus generic medications. There could be significant differences between the two, and this is an issue which should be studied in future research.

Based on the material available for analysis, no direct evaluation of the severity or length of shortages could be made. It is possible that shortages affecting all equivalent products and/or having high clinical significance have somewhat different characteristics than shortages affecting only one manufacturer and/or having only minor clinical significance. There might also be differences between brief and long duration shortages, including differences in root causes that are relevant for future actions to prevent and/or mitigate.

### Policy implications

The convergence of a pandemic, a destabilizing military conflict, and overly dependent supply chains make sustainable access to medicines a strategic priority. Policies to achieve a reliable supply of essential medicines must, however, include more than shortage registers. Preventive management requires coordinated policies to proactively address shortage risks and maintain sufficient stockpiles.

Looking beyond the boundaries of the current research to the larger context of drug shortages at this moment in time will require (i) enlightened medicines policies, (ii) a focus on implementation and coordination, and (iii) research to improve the effectiveness of these policies.

## Conclusions

Public drug shortage registers are vital data sources for proactively maintaining and managing a reliable drug supply. However, our study demonstrates that much work remains to standardize the contents and quality of public register data.

Drug shortages seem to vary between countries in terms of number of shortages, therapeutic use and formulation of the medicines involved, and possibly the price level of the medicines. These findings indicate that the shortages may not be solely a consequence of manufacturing disruptions but may reflect other contributing factors in the international drug distribution and supply mechanisms, including price differences and profit margins between national pharmaceutical markets.

Many questions remain concerning how market mechanisms and structures, regulatory approaches, and health system precautions might affect the occurrence and resolution of drug shortages. Further research should focus on implementation strategies to inform policymakers who want to solve rapidly increased shortage problems. Increased international cooperation is needed. If economic reasons are driving drug shortages, effective economic sanctions and incentives by healthcare providers and public actors should be considered to ensure a continuous and equitable supply of at least essential medicines.

## Supplementary Information

**Additional file 1.**

**Additional file 2.**

**Additional file 3.**

## Data Availability

Original data is public and obtainable from Finnish Medical Agency, Swedish Medical Products Agency, Norwegian Medicines Agency, Spanish Agency of Medicines and Medical Devices and United States Food and Drug Administration. The datasets analysed during the current study are also available upon reasonable request to the corresponding author. Data used is available from national medicines authorities: Finnish Medicines Agency (Fimea), Swedish Medical Products Agency, Norwegian Medicines Agency, Spanish Agency for Medicines and Health Products (AEMPS), Food and Drug Administration (FDA).

## Abbreviations

ATC: Anatomical Therapeutic Chemical
EEA: European Economic Area (European Union, Norway, Iceland, Lichtenstein)
FDA: Food and Drugs Administration
EMA: European Medicines Agency
WHO: World Health Organisation
OTC: Over-the-counter
