# Supplementary material for: National and transnational drug shortages: a quantitative descriptive study of public registers in Europe and the USA
Source: BMC Health Serv Res. 2022 Jul 22;22:940. doi: 10.1186/s12913-022-08309-3 (PMC9306441; doi:10.1186/s12913-022-08309-3)
Supplement: Supplementary file 1 — Additional file 1. [file 12913_2022_8309_MOESM1_ESM.docx]

Appendix 1

**European shortage registers**

Online shortage registers listed by European Medicines Agency (October 2020) and investigated in the study:

| Austria | [https://medicineshortage.basg.gv.at](https://medicineshortage.basg.gv.at/) (DE) |
| --- | --- |
| Belgium | [https://www.famhp.be](https://banquededonneesmedicaments.fagg-afmps.be/) (EN) |
| Bulgaria | [http://www.bda.bg/bg/](https://sespa.mh.government.bg/sespa/f?p=100:1:0:::::) (BG) |
| Croatia | [http://www.halmed.hr/en](http://www.halmed.hr/en/Promet-proizvodnja-i-inspekcija/Promet/Prekid-opskrbe-trzista-lijekom-i-nestasice/) (EN) |
| Czechia | [http://www.sukl.cz](http://www.sukl.cz/vypadky-leku) (CZ) |
| Denmark | [https://laegemiddelstyrelsen.dk](https://laegemiddelstyrelsen.dk/da/godkendelse/kontrol-og-inspektion/mangel-paa-medicin/meddelelser-om-forsyning-af-medicin/) (DK) |
| Estonia | [http://www.ravimiamet.ee](https://www.ravimiregister.ee/en/publichomepage.aspx) (ET) |
| Finland | [http://www.fimea.fi](https://www.fimea.fi/web/en/databases_and_registers/shortages) (FI) |
| France | [https://ansm.sante.fr](https://ansm.sante.fr/S-informer/Informations-de-securite-Ruptures-de-stock-des-medicaments) (FR) |
| Germany | [https://www.bfarm.de](https://www.bfarm.de/DE/Arzneimittel/Arzneimittelzulassung/Arzneimittelinformationen/Lieferengpaesse/_functions/Filtersuche_Formular.html?nn=11296612) (DE) / [www.pei.de](http://www.pei.de/lieferengpaesse-impfstoffe-human) (DE) |
| Greece | [http://www.eof.gr](http://www.eof.gr/web/guest/eparkeia) (GR) |
| Hungary | [https://www.ogyei.gov.hu](https://www.ogyei.gov.hu/gyogyszeradatbazis/) (HU) |
| Ireland | [https://www.hpra.ie/](https://www.hpra.ie/homepage/medicines/medicines-information/medicines-shortages) (EN) |
| Italy | <https://www.aifa.gov.it/en/web/guest/farmaci-carenti> (IT) |
| Latvia | [https://www.zva.gov.lv](https://www.zva.gov.lv/lv/zalu-piegades-partraukumu-parvaldiba-latvija) (LV) |
| Lithuania | [http://vvkt.lt/lit](https://www.vvkt.lt/index.php?2644926549) (LT) |
| Romania | [https://www.anm.ro](https://www.anm.ro/medicamente-de-uz-uman/autorizare-medicamente/notificari-discontinuitate-medicamente/) (RO) |
| Slovakia | [https://www.sukl.sk](https://portal.sukl.sk/PreruseniePublic/?act=PrerusenieOznList&mId=2&goto=1) (SK) |
| Slovenia | [http://www.jazmp.si](http://www.jazmp.si/humana-zdravila/podatki-o-zdravilih/zdravila-na-trgu/) (SI) |
| Spain | [https://www.aemps.gob.es](https://www.aemps.gob.es/distribucion-de-medicamentos/problemas-de-suministro-de-medicamentos/) (EN) |
| Sweden | [https://lakemedelsverket.se](https://lakemedelsverket.se/OVRIGA-SIDOR/Restnoteringar/) (SE) |
| Iceland | [https://www.lyfjastofnun.is](https://www.lyfjastofnun.is/lyf/lyfjaskortur/lyfjaskortsfrettir/) (IS) |
| Norway | [www.noma.no](https://legemiddelverket.no/legemiddelmangel/legemiddelmangel-og-avregistreringer-2017-rad-til-apotek-og-helsepersonell) (NO) |

**Registers not found (3):**

Estonia, Romania, Lithuania

**Published notification data incomplete (9):**

Austria, Belgium, Bulgaria, Denmark, Finland*, France, Germany, Ireland, Italy

**Notification data not including ATC codes (8):**

Croatio, Czech, Greece, Iceland, Latvia, Slovakia, Slovenia, Spain*****

**Notification data seriously underreported (1)**

Hungary

**Registers included in the study:**

Finland*, Spain*, Norway, Sweden, USA

**country included in the final selection*
