# Supplementary material for: National and transnational drug shortages: a quantitative descriptive study of public registers in Europe and the USA
Source: BMC Health Serv Res. 2022 Jul 22;22:940. doi: 10.1186/s12913-022-08309-3 (PMC9306441; doi:10.1186/s12913-022-08309-3)
Supplement: Supplementary file 2 — Additional file 2. [file 12913_2022_8309_MOESM2_ESM.docx]

**Appendix 2. Price comparison of US and Finnish cream and gel products**

There were 60 shortage notifications referring to creams and gels in the Finnish shortage register. We excluded those referring to OTC products, which left 41 products in 25 ATC ingredient categories.

Comparison was made by ingredient categories and closest equivalents were used in terms of strength, exact form (gel, lotion or cream) and package size.

Price data was searched from US GoodRx retail pharmacy price service (goodrx.com) and Finnish Yliopiston Apteekki (University Pharmacy, yliopistonapteekki.fi) net pages. All price data was retrieved December 7^th^, 2020.

Of 25 ATC ingredient categories, 6 were excluded, because there were OTC equivalents, although cases themselves referred to prescription products. Three (3) products were excluded, because 1) no equivalent product was found from GoodRx service and 2) because the product was a hospital product, and 3) no price information was found from GoodRx service.

Finnish retail drug prices are regulated and uniform in all pharmacies. In the USA, price competition exists, and prices vary. For comparison, the lowest price given by service was used, which means price lower than average.

For currency, a rate of 1.15 dollars/euro was used. Finnish prices were corrected 9% downwards to reflect the effect of 10% sales tax which was included in prices given by service. In the USA sales tax is normally not charged for prescription drugs and it was assumed not to be included in the price.

*Appendix 2: Table 1.*

| **Product** | **Retail price US ($) GoodRx, /g** | **Retail price FI (€) YA, /g** | **Price ratio (currency and VAT corrected)** |  |
| --- | --- | --- | --- | --- |
| Clobetasol 0.5% | 0.34 | 0.16 | 203% |  |
| Tacrolimus 0.1% | 1.61 | 0.69 | 223% |  |
| Metronidazole 1% | 1.15 | 0.43 | 256% |  |
| Metronidazole 0.75% | 0.81 | 0.34 | 228% |  |
| Desonide 0.1% | 0.79 | 0,17 | 444% | US price, for 0.05% strength |
| Betamethasone 0.1% | 0.46 | 0,16 | 275% |  |
| Mometasone 0.1% | 0.46 | 0.14 | 314% |  |
| Testosterone 2% | 0.47 | 0.74 | 61% | US price for 1.62% strength |
| Mupirosine 2% | 4.64 | 5.60 | 79% |  |
| Calcitriol 3mcg/g | 2.72 | 0.31 | 838% |  |
| Adapalene 0.1% | 0.73 | 0.36 | 194% |  |
| Adapalene/benzoyl peroxide 0.1%/2.5% | 0.88 | 0.78 | 108% |  |
| Azelaic acid 15% | 0.9 | 0.4 | 215% |  |
| Ivermectin 1% | 4.02 | 0.94 | 409% |  |
| Clindamycin 1% | 0.57 | 0.93 | 59% |  |
| **Mean price ratio** |  |  | **260%** |  |
| **Median price ratio** |  |  | **223%** |  |
