# Supplementary material for: National and transnational drug shortages: a quantitative descriptive study of public registers in Europe and the USA
Source: BMC Health Serv Res. 2022 Jul 22;22:940. doi: 10.1186/s12913-022-08309-3 (PMC9306441; doi:10.1186/s12913-022-08309-3)
Supplement: Supplementary file 3 — Additional file 3. [file 12913_2022_8309_MOESM3_ESM.docx]

Appendix 3.

Appendix 3: Table 1. Distribution of shortage notifications (n=5132) by ATC main class and country

| ATC class |  | Spain | Finland | Norway | Sweden | USA | Total |
| --- | --- | --- | --- | --- | --- | --- | --- |
| N: Nervous system | n | 227 | 406 | 203 | 227 | 553 | 1616 |
|  | % | 27.9% | 26.7% | 25.4% | 25.5% | 50.0% | 31.5% |
| C: Cardiovascular | n | 123 | 264 | 83 | 88 | 66 | 624 |
|  | % | 15.1% | 17.3% | 10.4% | 9.9% | 6.0% | 12.2% |
| A: Alimentary tract and metabolism | n | 61 | 140 | 71 | 91 | 76 | 439 |
|  | % | 7.5% | 9.2% | 8.9% | 10.2% | 6.9% | 8.6% |
| J: Antiinfective for systemic use | n | 85 | 72 | 58 | 57 | 115 | 387 |
|  | % | 10.4% | 4.7% | 7.3% | 6.4% | 10.4% | 7.5% |
| L: Antineoplastic and immunomodulating | n | 102 | 108 | 56 | 58 | 57 | 381 |
|  | % | 12.5% | 7.1% | 7.0% | 6.5% | 5.2% | 7.4% |
| G: Genito urinary system and sex hormones | n | 42 | 124 | 60 | 65 | 0 | 291 |
|  | % | 5.2% | 8.1% | 7.5% | 7.3% | 0.0% | 5.7% |
| M: Musculo-skeletal system | n | 29 | 69 | 71 | 42 | 78 | 289 |
|  | % | 3.6% | 4.5% | 8.9% | 4.7% | 7.1% | 5.6% |
| R: Respiratory system | n | 10 | 133 | 38 | 59 | 13 | 253 |
|  | % | 1.2% | 8.7% | 4.8% | 6.6% | 1.2% | 4.9% |
| B: Blood and blood forming organs | n | 35 | 79 | 17 | 48 | 37 | 216 |
|  | % | 4.3% | 5.2% | 2.1% | 5.4% | 3.3% | 4.2% |
| D: Dermatologicals | n | 36 | 46 | 52 | 38 | 1 | 173 |
|  | % | 4.4% | 3.0% | 6.5% | 4.3% | 0.1% | 3.4% |
| H: Hormones, excluding sex hormones | n | 38 | 33 | 30 | 57 | 11 | 169 |
|  | % | 4.7% | 2.2% | 3.8% | 6.4% | 1.0% | 3.3% |
| S: Sensory organs | n | 10 | 30 | 39 | 32 | 47 | 158 |
|  | % | 1.2% | 2.0% | 4.9% | 3.6% | 4.2% | 3.1% |
| V: Various | n | 10 | 16 | 20 | 21 | 34 | 101 |
|  | % | 1.2% | 1.1% | 2.5% | 2.4% | 3.1% | 2.0% |
| P: Antiparasitic | n | 6 | 2 | 2 | 7 | 18 | 35 |
|  | % | 0.7% | 0.1% | 0.3% | 0.8% | 1.6% | 0.7% |
| Total | n | 814 | 1522 | 800 | 890 | 1106 | 5132 |
|  | % | 100.0% | 100.0% | 100.0% | 100.0% | 100.0% | 100.0% |

Appendix 3: Table 2. Distribution of shortage notifications by drug formulation and country (n=5132)

| Drug formulation |  | Spain | Finland | Norway | Sweden | USA | Total |
| --- | --- | --- | --- | --- | --- | --- | --- |
| Tablets | n | 336 | 752 | 321 | 327 | 332 | 2068 |
|  | % | 41.3% | 49.4% | 40.1% | 36.7% | 30.0% | 40.3% |
| Injectables | n | 253 | 258 | 145 | 270 | 635 | 1560 |
| (1) | % | 31.1% | 17.0% | 18.1% | 30.3% | 57.4% | 30.4% |
| Capsules | n | 57 | 105 | 41 | 45 | 75 | 323 |
|  | % | 7.0% | 6.9% | 5.1% | 5.1% | 6.8% | 6.3% |
| Modified tablets | n | 32 | 97 | 48 | 47 | 8 | 232 |
| (2) | % | 3.9% | 6.4% | 6.0% | 5.3% | 0.7% | 4.5% |
| Gels/Creams | n | 37 | 60 | 48 | 43 | 0 | 188 |
|  | % | 4.5% | 3.9% | 6.0% | 4.8% | 0.0% | 3.7% |
| Ophthalmic | n | 11 | 27 | 38 | 25 | 46 | 147 |
|  | % | 1.4% | 1.8% | 4.8% | 2.8% | 4.2% | 2.9% |
| Solutions | n | 22 | 31 | 30 | 27 | 7 | 117 |
|  | % | 2.7% | 2.0% | 3.8% | 3.0% | 0.6% | 2.3% |
| Modified capsules | n | 10 | 18 | 12 | 5 | 0 | 45 |
| (3) | % | 1.2% | 1.2% | 1.5% | 0.6% | 0.0% | 0.9% |
| Other | n | 56 | 174 | 117 | 101 | 3 | 451 |
| (4) | % | 6.9% | 11.4% | 14.6% | 11.3% | 0.3% | 8.8% |
| Total | n | 814 | 1522 | 800 | 890 | 1106 | 5132 |
|  | % | 100.0% | 100.0% | 100.0% | 100.0% | 100.0% | 100.0% |

1)- I.V.and I.M. injection and infusion products, dialysis products

2)- Depot tablets, extended-release tablets

3)- Depot capsules, extended-release capsules

4) -Suppositories, dermal patches, inhalation products, implants etc.
